# Motivation to interaction media: The impact of automation trust and self-determination theory on intention to use the new interaction technology in autonomous vehicles

**DOI:** 10.3389/fpsyg.2023.1078438

**Published:** 2023-02-08

**Authors:** Yubin Xie, Ronggang Zhou, Alan Hoi Shou Chan, Mingyu Jin, Miao Qu

**Affiliations:** ^1^School of Economics and Management, Beihang University, Beijing, China; ^2^Department of Advanced Design and Systems Engineering, City University of Hong Kong, Hong Kong, China; ^3^Key Laboratory of Complex System Analysis, Management and Decision (Beihang University), Ministry of Education of the People's Republic of China, Beijing, China

**Keywords:** self-determination theory, motivation, autonomous vehicles, automation trust, interaction technology

## Abstract

**Introduction:**

This research investigated the effects of three psychological needs (competence, autonomy, and relatedness) of self-determination theory (SDT) and automation trust on the intention of users to employ new interaction technology brought by autonomous vehicles (AVs), especially interaction mode and virtual image.

**Method:**

This study focuses on the discussion from the perspective of psychological motivation theory applied to AV interaction technology. With the use of a structured questionnaire, participants completed self-report measures related to these two interaction technologies; a total of 155 drivers’ responses were analyzed.

**Result:**

The results indicated that users’ intentions were directly predicted by their perceived competence, autonomy, and relatedness of SDT and automation trust, which jointly explained at least 66% of the variance in behavioral intention. In addition to these results, the contribution of predictive components to behavioral intention is influenced by the type of interaction technology. Relatedness and competence significantly impacted the behavioral intention to use the interaction mode but not the virtual image.

**Discussion:**

These findings are essential in that they support the necessity of distinguishing between types of AV interaction technology when predicting users’ intentions to use.

## 1. Introduction

Driven by technology, the intelligent vehicle industry has developed rapidly, and the technology of intelligent assistants in autonomous vehicles (AVs) has gradually matured. Google has been promoting driverless development since 2009 (Lutin et al., 2013). In 2015, Mercedes-Benz driverless trucks were licensed and began road testing (Bimbraw, 2015). In the same year, Baidu’s innovative vehicles achieved fully autonomous driving in urban, ring road, and highway road conditions (Ziyan and Shiguo, 2021). The United Kingdom government began testing driverless cars on freeways in 2017 and made self-driving vehicles on the road in 2020 (Ryan, 2020). With the commercialization of 5G and the continuous development of artificial intelligence technology, intelligent vehicles have developed greatly. Some new interaction technology brought by intelligent vehicles has attracted people to use them (Hensher, 2018).

Vehicle-to-X/Vehicle-to-everything is critical technology for the intelligent interactions of AVs, which involve vehicle-to-outside information exchange (Lu et al., 2014). Telematics lays down a new direction of automotive technology development by integrating global positioning system (GPS) navigation technology, vehicle-to-vehicle communication technology, wireless communication, and remote sensing technology to achieve compatibility between manual driving and autonomous driving. It enables communication between cars, between base stations, and between cars and base stations. Thus, a series of traffic information, such as real-time road conditions, road information, and pedestrian information, can be obtained to improve driving safety, reduce congestion, improve traffic efficiency, and provide in-car entertainment information. The interaction in V-X can be divided into two main categories: 1. the function related to the interaction technology itself - the interaction mode; and 2. the carrier of interaction technology - the virtual image (Fuyun et al., 2019; Liu and Liu, 2021). The interaction mode includes the information, tools, and channels on which a specific interaction behavior relies and the logic by which that interaction behavior is realized. The design of the interaction mode affects the fluency of human-vehicle communication. The virtual image is a space for interaction manifestation, performance, and confession. Virtual image design is inseparable from vehicle owners’ characteristics (Li et al., 2021). Different styles of virtual images affect personal preference and love for interaction differently.

As the importance of automotive software continues to rise, traditional interaction mediums are ushering in new ways of interacting (Sotolongo and Copulsky, 2018). Operations such as switching on/off the air conditioner, playing/stopping music, and opening/closing the windows in traditional vehicles are being reimplemented through new intelligent interaction modes. An increasing number of scholars claim that the interaction process with AVs should be closer to natural communication between humans. Naturalized interaction behavior is more helpful for the human understanding of products. It increases the sense of integration and realism in product use (Wang, 2021), reducing the cognitive burden and improving interaction efficiency. The establishment of multichannel interaction based on touch, voice, air action, and expression can enhance the interaction between humans and information and provide a more three-dimensional interaction experience for users. The inspirational design makes interaction more human and natural, providing emotional support and rich interaction channels, which is especially meaningful for people to understand autonomous driving systems’ behaviors (Murali et al., 2022).

Changes in intelligent vehicle technologies have raised new questions. We have given smarter functions to machines and added human attributes to them. This has led to a very different route for developing smart technologies than traditional technology. The human-vehicle interaction model has changed from product use to human–machine codriving (Tan et al., 2021). In the past, users cared about only using the product, whether it was good or not, and whether it could achieve the expected function. The relationship between people and products is the relationship between using and being used. Now, the relationship established between people and intelligent products has converged, like interpersonal relationships (Zheng et al., 2019). Intelligent products need to meet not only the basic needs of users but also their emotional needs.

In this context, it is meaningful to discuss human intentions for AV interaction technology from a motivational perspective. It has been suggested that motivation has a multidimensional structure: intrinsic and extrinsic (Ryan and Deci, 2000a,b). Intrinsic motivation is motivation to use the product in terms of the user’s enjoyment. Extrinsic motivation involves using a product because of some external factor that is desirable for users (Grouzet et al., 2005). The technology acceptance model (TAM) is important for how technological innovation changes our consumption, acceptance, and use. In the TAM model, behavioral intentions are influenced by attitudes, which can be considered subjective manifestations of motivation (Davis, 1989). Müller (2019) used the PLS-SEM method to validate the TAM for AVs. They found the compound effect of personal experience and technological norms on technology acceptance by investigating three market groups in Europe, North America, and China. Yuen et al. (2021) examined the factors influencing users’ behavioral intentions to use AVs based on an integrated model of innovation diffusion theory (IDT) and TAM. The positive impact of perceived usefulness (PU) and perceived ease of use (PEOU) on users’ behavioral intentions to use AVs was confirmed. Furthermore, Seuwou et al. (2020) combined the TAM model and UTAUT theory of AVs by considering additional internal and external motivations such as self-efficacy, perceived safety, anxiety, and legal regulation. The above studies provide a good explanation of users’ behavioral intentions toward AVs, but they focus mainly on human extrinsic motivation (EM) theories and rarely adopt intrinsic motivation (IM) theories such as SDT. Intrinsic motivation is more explanatory and stable than extrinsic motivation, and intrinsic motivation is relatively stable over time. It is meaningful to discuss users’ behavioral intentions toward new interaction technologies for AVs from the perspective of intrinsic motivation theory. Self-determination theory (SDT) is based on the view that human motivation is related to the basic psychological needs of competence, autonomy, and relatedness. In more detail, the theory suggests that satisfying these three needs enhances intrinsic motivation and supports the internalization and integration of extrinsic motivation. SDT is also considered to be a key theory of intrinsic motivation theory. Therefore, adopting SDT to discuss this issue would make a significant contribution to advancing the literature.

### 1.1. Self-determination theory to understand user intentions

SDT is a macro theory of human motivation and personality that focuses on people’s intrinsic growth tendencies and innate psychological needs. It focuses on the reasons behind people’s choices (Ryan and Deci, 2000a,b; Deci and Ryan, 2012; Ryan and Deci, 2017). It recognizes the importance of the interrelatedness of intrinsic and extrinsic motivation as motivational means to achieve goals (Hergenhahn and Henley, 2013). It also suggests that people have innate psychological needs, which are the basis for self-motivation (Koole et al., 2019). In the theory of SDT, it is widely considered that the three primary intrinsic needs, autonomy, competence, and relatedness, play an important role in explaining people’s psychological needs (Deci and Ryan, 1991).

There is much evidence for the positive role of SDT in predicting the willingness to use smart technologies. Schaefer et al. (2014) first extended self-determination theory to the field of human factors, developed a theoretical model of motivation in human-technology interactions, and pointed out that satisfying the basic psychological needs (BPNs) of individuals can enhance user motivation. Villalobos-Zúñiga and Cherubini (2020) made self-determination theory useful in classifying and designing the layout of self-motivated applications (APPs). Research on predicting students’ behavioral intentions to use open-source software, which combined the views of the TAM and SDT, found an indirect impact of SDT on behavioral intention (Racero et al., 2020). Regarding interaction technology, Rupp et al. (2016) found that people’s motivation to use fitness products is influenced by the extent to which they meet basic psychological needs. The study of human-computer interaction games reached a similar conclusion (Tyack and Mekler, 2020). Yang and Aurisicchio (2021) used self-determination theory to design conversational agents.

Regarding virtual technology, Jung’s (2011) study examined user experiences in virtual social worlds, demonstrating that the psychological need for perceived autonomy was related to user satisfaction and subsequently predicting that users would continue to return to virtual social worlds. Hoffman and Novak (2012) argued that virtual worlds provide individuals with opportunities to satisfy basic psychological needs such as autonomy, competence, and relatedness, which influence customers’ motivational behaviors. Research investigating motivation and experience in virtual learning environments revealed significant associations among perceived autonomy, competence, relatedness and intrinsic motivation (Huang et al., 2019). The research on predicting the acceptance of MOOCs, which is also a virtual platform, found a positive relationship between the composition of SDT and intention.

In autonomous vehicles, many studies have mentioned the role of psychological ownership and self-efficacy (Acheampong and Cugurullo, 2019; Lee et al., 2019), which has similar characteristics to SDT. Few studies have used SDT to predict behavioral intentions for autonomous use, especially for interaction technology related to AVs (Rupp et al., 2016). However, previous research in other areas is sufficient to demonstrate the relationship between SDT and behavioral intention. Therefore, this study predicts that the psychological needs of autonomy, competence, and relatedness positively affect the behavioral preferences of AVs’ interaction modes and virtual images. Based on the above research, we propose the following hypotheses:

*H1*: Perceived autonomy is positively associated with the behavioral intentions toward AVs’ interaction mode and virtual images.

*H2*: Perceived competence is positively related to the behavioral intentions toward AVs’ interaction mode and virtual images.

*H3*: Perceived relatedness is positively linked to the behavioral intentions toward AVs’ interaction mode and virtual images.

### 1.2. The role of automation trust in predicting user intentions

In addition to focusing on intelligent interaction to satisfy users’ psychological needs, we need to focus on the basic use of the product function. In the research on autonomous vehicle acceptance, automation trust is an important factor influencing the willingness to use automated systems in several domains. Automation trust is a human attitudinal judgment of the degree to which users can rely on an automated system to achieve a specific goal in an uncertain situation. How humans appropriately estimate trust in a system is key to the safety and efficiency of human-computer intelligence collaboration (Parasuraman and Riley, 1997). Lee and See (2004) opened a chapter in the study of the impact of automation trust by considering the factors that influence trust and the role of trust in regulating dependence on automation based on the theory of reasoned action. They proposed a conceptual model of trust management and the dynamic process of trust’s impact on dependence. Schaefer et al. (2014) proposed the concept of robot trust and developed a three-factor model of trust in automation based on the “human-robot” trust model. The model considers that human, automation, and environmental factors jointly influence automation trust. In the autonomous vehicle research field, some scholars have correlated automation trust and willingness in their studies and have obtained enlightening findings. A study of social media uses by Wang and Li (2014) also noted the effect of trust on the acceptance of recommended content on social media.

Automation trust is widely studied in autonomous driving to predict people’s acceptance of and willingness to use AVs. They supported the claim that trust is the main factor influencing the acceptance of AVs (Choi and Ji, 2015; Adnan et al., 2018). Bazilinskyy et al. (2015) supported the findings by referring to the partial distrust of the autonomous vehicles by those who prefer manual or partially automated driving to fully automatic driving. Again, this suggests that a high level of trust is a barrier that needs to be overcome for users to accept self-driving vehicles. Meanwhile, Bansal and Kockelman (2017) concluded that older people are less willing to use AVs, which could be due to trust issues. Kaur and Rampersad (2018) found that automation trust positively correlated with adopting AVs. In addition to trust in autonomous vehicles, much research focuses on independent vehicle interaction systems. Long et al. (2020) found that personalization can increase trust in automation and thus increase user willingness to use the system by accepting and understanding each driver’s behavior. Anthropomorphic messages have been shown to promote the perception of AVs as social subjects and to enhance trust in these cars (Niu et al., 2018). However, there is less directly relevant literature demonstrating the effect of automation trust on the behavioral intention for specific interaction technologies such as interaction mode and virtual image. Research on trust in technology has shown that trust in AVs directly affects the intention to adopt such vehicles. Therefore, we propose the following hypothesis.

*H4*: Automation trust is positively linked to the behavioral intentions toward AVs’ interaction mode and virtual image.

### 1.3. Mediating effect linking SDT, automation trust, and behavioral intention

After clarifying the relationship between automation trust and behavioral intention, we would like to explore the relationship between SDT and automation trust, clarifying the mediating effect of automation trust. Rupp et al. (2016) conducted a study on the effects of user characteristics (i.e., personality, age, computer self-efficacy, physical activity level) and device characteristics (trust, usability, and motivational burden) on behavioral intention to use wearable fitness devices. They found that people’s trust to fitness equipment is effective motivates them to achieve their fitness goals, while trust also has a significant effect on perceived motivational affordances. Users need to believe that the information is trustworthy for using fitness equipment to motivate them to become more active. Although there is no more direct evidence on the direct effect of the three psychological needs of SDT on automation trust, there is some indirect evidence on the effect of SDT on trust. Hew et al. (2017) demonstrated the influence of the three psychological needs of SDT on website trust and clarified the mediating role of trust in the relationship between SDT and behavioral intention. In addition, some interpretations and conjectures have been made from the perspective of understanding the three psychological needs.

Autonomy typically describes the need for people to feel that their behavior is their choice. Deci et al. (1989) found that managers give more autonomy to their laborers when they possess a higher degree of trust in the organization. At the same time, many studies demonstrate the positive effect of perceived behavioral control on automation trust (Buckley et al., 2018; Velasco et al., 2019; Zhang et al., 2019). From the perspective of autonomous vehicles, Rödel et al. (2014) found that the autonomy levels of vehicles influenced trust in the system. If a vehicle has a high level of autonomy, people have less trust in it. Other research has shown that the automation allocation logic influences the trust in AI in the social integration of artificial intelligence. When the level of automation matches the level of autonomy, the AI is trustworthy, and the whole system is balanced. Therefore, the hypothesis is theorized as follows:

*H5*: Perceived autonomy is positively associated with the automation trust in AVs’ interaction mode and virtual image.

Competence is usually defined as the user’s desire to feel competent and efficient in their interactions with the system. Barber (1983) identified three trust factors in social relations: competence, persistence, and fiduciary responsibility. Lee et al. (2019) noted that functional incompetence damages trust. In addition, from the view of the definition of competence, this definition and understanding have similar implications to usability testing SUS and the TAM or TPB models of ease of learning and perceived ease of use. Venkatesh et al. (2012) demonstrated the impact of perceived ease of use in the TAM on the automation trust of AVs. On the other hand, Sadi and Al-Khalifah (2012) found that perceived competence has a positive effect on trust. Research on trust in online transactions has also proven competence’s positive effect (Kim and Kim, 2005). Hence, the following hypothesis is posited:

*H6*: Perceived competence is positively related to the automation trust in AVs’ interaction mode and virtual image.

Relatedness is the innate human desire to be connected, to love and be loved, and to belong. Many studies have demonstrated that a good human–machine system relationship can enhance trust in automation systems (Lyons et al., 2019; Zhou et al., 2020). When the need for relatedness is satisfied, a stronger relationship between kindness and benevolence leads to an increase in trust. Kofi et al. (2020) also found that relatedness with social media brands influences trust in this brand. Based on the above considerations and discussions, we propose the following hypothesis:

*H7*: Perceived relatedness is positively linked to the automation trust in AVs’ interaction mode and virtual image.

### 1.4. Overview of studies

**Figure 1:**
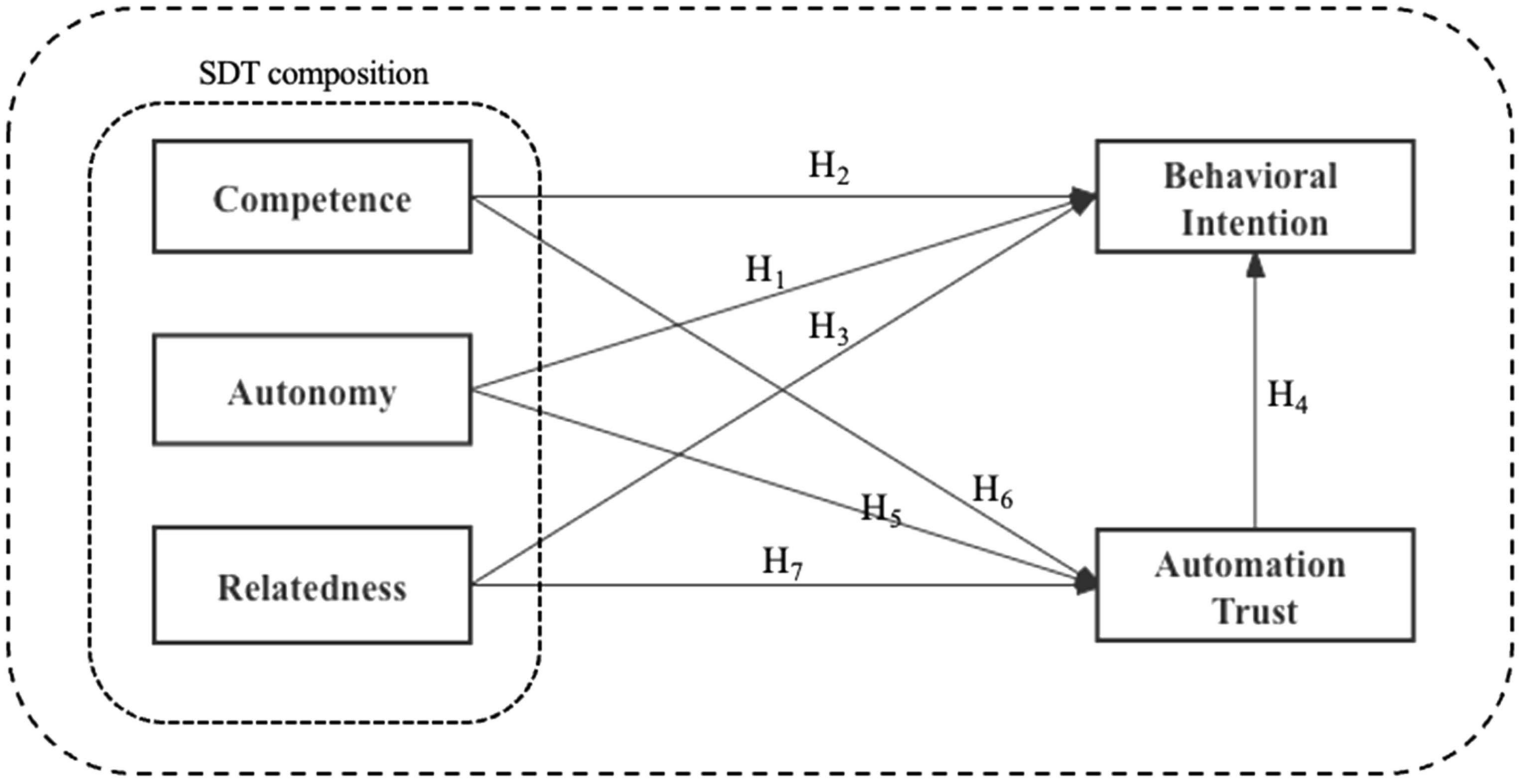
Proposed research model.

Based on this consideration, this study proposes a research model that integrates components of automation trust and revised SDT to examine the influence of factors on the behavioral intention of interaction modes and virtual images (shown as Figure 1). In addition, this work compares the predictive effects of the three basic psychological needs of SDT on the use of intelligent interaction modes and virtual images, specifically interpreting them considering their characteristics. The study results benefit autonomous vehicle designers and salespeople, helping them determine appropriate design and marketing strategies to improve driving safety and accident prevention performance and thus increase the acceptance of AVs in the future.

## 2. Materials and methods

### 2.1. Participants

A total of 155 drivers (87 males and 68 females) were recruited for the experiment, aged from 21 to 40 years old (mean = 27.93, SD = 4.79). All participants were recruited from local communities through the internet. To ensure the accuracy of the measurements and the concentration of the sample, we selected participants with Beijing driving experience as the study sample. All participants were required to have no less than 1 year of driving experience in Beijing, China. This study was reviewed and approved by the Human Research Ethics Committee of ***(Hidden author organization). All the drivers volunteered to participate in the experiment. At the end of the investigation, each participant received 20 or 30 CNY as compensation based on performance. The demographic profile of the participants is shown in Table 1.

**Table 1:** Basic information about the participants of this study.

Characteristics		Numbers	Proportion (%)
Gender	Male	87	56.13
	Female	68	43.87
Age	21–25	55	35.48
	26–30	59	38.06
	31–35	26	16.78
	36–40	15	9.68
Education	Junior High School	2	1.29
	High School	6	3.87
	Bachelor’s degree	99	63.87
	Master’s Degree	48	30.97
Driving experience	1–2 years	42	27.10
	3–4 years	52	33.55
	5–6 years	29	18.71
	Above 6 years	32	20.65
Autonomous experience	With	39	25.16
	Without	116	74.84

### 2.2. Materials

#### 2.2.1. Autonomous interaction technology

Previous research on interaction has mainly dealt with two aspects of exchange: the intrinsic mode of operation and the extrinsic mode of expression. Two interaction technologies arise in AVs: the interaction mode and the virtual image. One is the mechanism of action by which the user and the AV communicate, and the other is the vehicle by which the AV expresses their communication.

##### 2.2.1.1. Interaction mode

**Figure 2:**
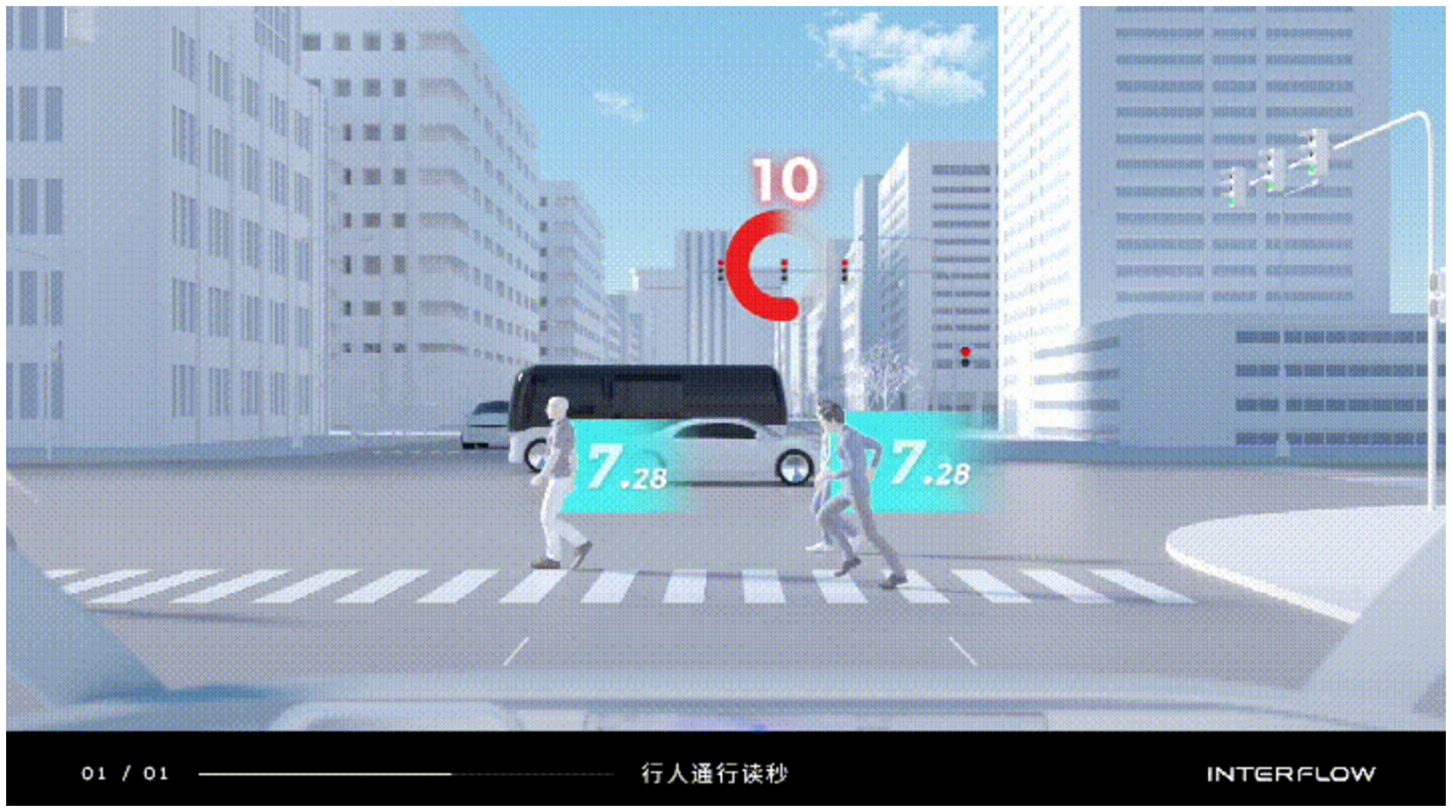
Video screenshot of the experimental material of the interaction mode.

The interaction mode is a specific interaction behavior that relies on the information, tools, channels, and logic of the interaction behavior to achieve. Examples include in-vehicle ordering, restaurant reservations, shopping, pedestrian crossing alerts, and other services through in-vehicle touch interactions, voice interactions, gesture interactions, and so on. In this study, we selected a *pedestrian crossing alert* status from the Baidu Apollo autonomous vehicle introduction video as the evaluation object of the interaction model. The *Pedestrian Crossing Alert* indicated that when the AV arrived at a crossing, the cockpit windshield displayed the waiting and the estimated time for pedestrians to pass. It allowed the driver to determine whether it was a dangerous situation requiring a takeover. We edited an introductory video about this interaction using iMovie in the experiment. By watching the video, the participants could clearly understand how the interaction was implemented. The conceptual picture of this interaction mode is shown in Figure 2.

##### 2.2.1.2. Virtual image

**Figure 3:**
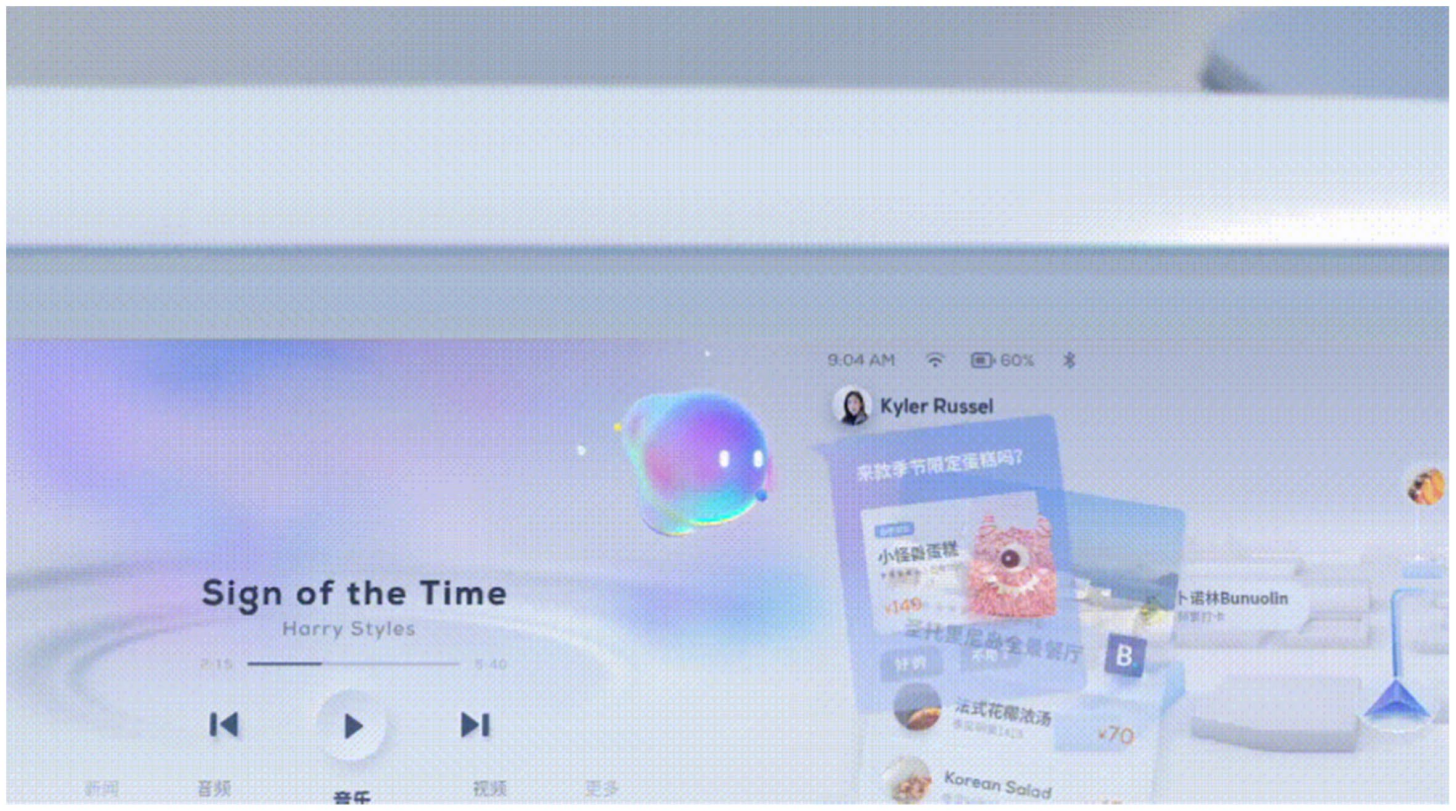
Video screenshot of the experimental material of the virtual image.

A virtual image is a medium of expression for interaction. We can consider it as the personification of the car, which turns human-vehicle functional use into communication. Humans, robots, and animals are the most common species in virtual images. In this study, we selected the *anthropomorphic virtual assistant* used in the Baidu Apollo autonomous vehicle as the evaluation object of the virtual image, as shown in Figure 3. The *anthropomorphic virtual assistant* allows for some movement in the interaction and eye contact with the user. The assistant can perform some functions like a human assistant and behave more dexterously. In the experiment, we edited an interaction video about this virtual assistant using iMovie. By watching the video, the participants can obtain an intuitive feeling for the virtual image.

### 2.3. Questionnaire

We developed a self-administered questionnaire including *competence, autonomy, and relatedness* from the SDT questionnaire; behavioral intention from the TAM questionnaire; and the automation trust questionnaire. There were two different questionnaires, one to evaluate the interaction mode and the other to evaluate the virtual image. They describe almost the same content, except for the change in the evaluation subjects.

#### 2.3.1. Behavioral intention

Behavioral intention was assessed by calculating the mean score of the following two items (Davis, 1989; Venkatesh and Davis, 2000; Chen et al., 2009): “I would like to use this interaction mode/virtual image” (BI1) and “I think I might use that interaction mode/virtual image” (BI2). These two questions were derived from the original TAM questionnaire (Davis, 1989), in which we replaced the evaluation subjects. The factor used a seven-point Likert scale that is consistent with the original scale, from “1” to “7,” representing “completely disagree” to “completely agree.” The participants were asked to complete a 7-point Likert-type scale ranging from totally disagree to totally agree (1 = I do not agree at all; 7 = I totally agree).

#### 2.3.2. Automation trust

Following a method used in previous studies (Jian et al., 2000; Buckley et al., 2018; Natarajan and Gombolay, 2020; Chiou and Lee, 2021), the mean score of the following questions was used to measure automation trust. All questions were derived from the automation trust scale (Jian et al., 2000), which has been validated. The specific description of the automation trust scale is shown in Table 2. The factor also used a seven-point Likert scale.

**Table 2:** The questions of the automation trust scale (Jian et al., 2000).

Items	Questions
AT1 (−)	The interaction mode/virtual image is deceptive
AT2 (−)	The interaction mode/virtual image behaves in an underhanded manner
AT3 (−)	I am suspicious of the interaction mode’s (virtual image) intent, action, or outputs
AT4 (−)	I am wary of the interaction mode/virtual image
AT5 (−)	The interaction mode’s (virtual image) actions will have a harmful or injurious outcome
AT6 (+)	I am confident in the interaction mode/virtual image
AT7 (+)	The interaction mode/virtual image provides security
AT8 (+)	The interaction mode/virtual image has integrity
AT9 (+)	The interaction mode/virtual image is dependable
AT10 (+)	The interaction mode/virtual image is reliable
AT11 (+)	I can trust the interaction mode/virtual image
AT12 (+)	I am familiar with the interaction mode/virtual image

#### 2.3.3. Competence, autonomy, and relatedness

*Competence, autonomy, and relatedness* are three parts of psychological demand in self-determination theory. After revising Peters’ *Autonomy and Competence in Technology Adoption Questionnaire* (ACTA), *Technology-based Experience of Need Satisfaction – Interface questionnaire* (TENS-Interface), and *Technology-based Experience of Need Satisfaction – Task questionnaire* (TENS-Task) (Peters et al., 2018), we initially constructed a series of questions to measure the three aspects of psychological demands: competence, autonomy, and relatedness. *Competence* was assessed by calculating the mean score of the three positive scales and three transformed negative scales. The specific description of the competence is shown in Table 3 (C1-C6). *Autonomy* was assessed by calculating the mean score of the two positive scales and six transformed negative scales. The specific description of autonomy is shown in Table 3 (A1-A5). *Relatedness* was assessed by calculating the mean score of the three positive scales and two transformed negative scales. The specific description of the relatedness is shown in Table 3 (R1-R5). The factors of *competence, autonomy, and relatedness* were measured using a five-point Likert scale that is consistent with the original scale that ranged from “1″ to “5″, representing “completely disagree” to “completely agree.” The participants were asked to complete a 5-point Likert-type scale ranging from totally disagree to totally agree (1 = I do not agree at all; 5 = I totally agree).

**Table 3:** The questions of the self-determination theory scale (Peters et al., 2018).

Items	Questions
C1 (+)	I feel very capable and effective at using the interaction mode/virtual image
C2 (+)	I feel confident in my ability to use the interaction mode/virtual image
C3 (+)	It is easy to use the interaction mode/virtual image
C4 (−)	I found the interaction mode/virtual image challenging to use
C5 (−)	I found interface and controls of the interaction mode/virtual image confusing
C6 (−)	It was not easy to use this interaction mode/virtual image
A1 (+)	The interaction mode/virtual image provides me with useful options and choices
A2 (+)	I can get the interaction mode/virtual image to do the things I want it to
A3 (−)	I feel pressured by the interaction mode/virtual image
A4 (−)	The interaction mode/virtual image feels intrusive
A5 (−)	The interaction mode/virtual image feels controlling
R1 (+)	The interaction mode/virtual image makes me feel connected to people, things, and events outside of me
R2 (+)	Using the interaction mode/virtual image helps me to establish or maintain satisfactory relationships with people, things, and events outside of me
R3 (+)	Using the interaction mode/virtual image makes me to feel part of a larger community
R4 (−)	I do not feel close to other users of the interaction mode/virtual image
R5 (−)	The interaction mode/virtual image does not support meaningful connections to others

### 2.4. Procedure

**Figure 4:**
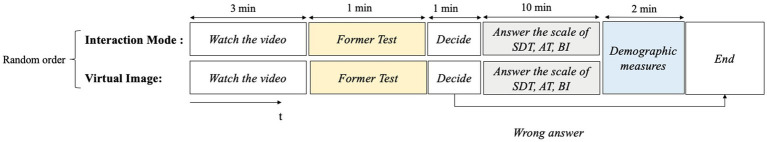
Procedure of the experiment per participant.

We performed the experiment using an online shared meeting and showed the videos using *WenjuanXing* (an e-scale collection platform). After recording demographic information, each driver was required to watch the introduction video of the *Interaction Mode* and the *Virtual Image*. After each video, they completed the trust in automation scale, the self-determination theory scale, and the behavioral intention scale once. The two videos were balanced in random order. To avoid being unfocused, we set up detailed test questions such as ‘How many interactions are presented in the video?’, which related to the content of the video to ensure that the driver watched it carefully. In these test questions, if the driver provided any wrong answer, the driver was disqualified from participating in further testing. There were also some discriminative questions, such as ‘Please select totally disagree with this question. If a driver provides an incorrect answer, they will receive a lower reward. If the driver provides more than one incorrect answer, the driver will be asked to quit the experiment. After evaluating the interaction mode and virtual image, the participants were asked to complete several demographic measures (e.g., age, gender, driving experience, autonomous experience). The procedures of our experiments are shown in Figure 4.

## 3. Results and analysis

### 3.1. Measurement model

We collected data from 155 participants, combining the two kinds of interaction technology for analysis, for a total of 310 data points. We used AMOS 21.0 to test the validity of the measurement model for each interaction technology using a five-factor structure including behavioral intention, automation trust, competence, autonomy, and relatedness. Following a similar previous study (e.g., Jung et al., 2009), we selected eight common model-fit metrics to estimate the fit of the measurement model. As shown in Table 4, although the value of χ^2^/df is larger than 3, it is less than 5, which is considered acceptable for both models. We also noticed that the CFI, NFI, and SRMR all reached an acceptable level. Therefore, we concluded that the collected data of the measurement model had an acceptable fitness level.

**Table 4:** Fit indices for the measurement model.

Fit indices	Recommended value	Interaction mode	Virtual image
χ^2^/df (chi-square/degrees of freedom)	<5	3.534	3.056
CFI (comparative fit index)	>0.9	0.961	0.922
RMSEA (root mean square error of approximation)	<0.1	0.098	0.096
SRMR (standardized root mean square residual)	<0.1	0.096	0.073
NFI (normed fit index)	>0.9	0.950	0.959

In addition to the model-fit effect, we also tested the measures’ validity and reliability for the interaction mode and virtual image by exploratory factor analysis (EFA), confirmatory factor analysis (CFA), and reliable test. In the first round of testing, we found that question Items C4, A8, R4, and R5 did not pass the validity test. After removing these question items from the second round, all question items passed the validity test. The results are shown in Table 5.

**Table 5:** Standardized factor loadings, AVE, CR and Cronbach’s alpha for the core variable questionnaires.

Construct	Item	Interaction mode (*n* = 155)				Virtual image (*n* = 155)			
		Item loading	CR	AVE	Cronbach’s alpha	Item loading	CR	AVE	Cronbach’s alpha
Behavioral intention	BI1	0.917	0.882	0.790	0.877	0.953	0.880	0.787	0.873
	BI2	0.859				0.816			
Automation trust	AT1	0.734	0.939	0.568	0.938	0.796	0.951	0.621	0.949
	AT2	0.615				0.759			
	AT3	0.710				0.809			
	AT4	0.757				0.760			
	AT5	0.690				0.764			
	AT6	0.774				0.804			
	AT7	0.773				0.743			
	AT8	0.768				0.700			
	AT9	0.878				0.856			
	AT10	0.873				0.873			
	AT11	0.862				0.839			
	AT12	0.534				0.732			
Competence	C1	0.655	0.836	0.506	0.831	0.743	0.862	0.557	0.859
	C2	0.717				0.680			
	C3	0.701				0.703			
	C5	0.679				0.813			
	C6	0.799				0.784			
Autonomy	A1	0.529	0.789	0.540	0.860	0.647	0.881	0.600	0.875
	A2	0.532				0.707			
	A3	0.646				0.801			
	A4	0.788				0.854			
	A5	0.829				0.843			
Relatedness	R1	0.797	0.887	0.725	0.887	0.803	0.884	0.718	0.883
	R2	0.895				0.842			
	R3	0.859				0.894			

For the interaction mode, the KMO was 0.927, the item loadings ranged from 0.529 to 0.917, and the cumulative variance explanation rate was 71.87%. The lowest average variance extracted (AVE) value among all components was 0.506, and the lowest CR was 0.789. For the virtual image, the KMO was 0.942, the item loadings ranged from 0.647 to 0.953, and the cumulative variance explanation rate was 74.50%. The lowest AVE value was 0.557, and the lowest CR was 0.880. If KMO > 0.8, item loading >0.4, and cumulative variance explanation rate > 50%, the questionnaire items had high convergent validity (Tabachnick et al., 2007; Comrey and Lee, 2013). According to Fornell and Larcker (1981), if the AVE value is higher than 0.50 and the composite reliability (CR) value is higher than 0.70, then the convergent validity is acceptable. Table 6 shows that all HTMT values were less than 0.9, indicating that the discriminant validity was acceptable (Hair Jr et al., 2021). All the Cronbach’s alpha values were over 0.8, which indicates that the scales had good reliability. In general, the reliability of the questionnaire for each interaction technology was acceptable.

**Table 6:** The heterotrait-monotrait ratio result.

Variable	1	2	3	4	5
Interaction mode (*n*=155)					
1. Behavioral intention	-	0.823	0.789	0.844	0.782
2. Automation trust		-	0.834	0.864	0.694
3. Competence			-	0.890	0.596
4. Autonomy				-	0.569
5. Relatedness					-
Virtual Image (*n*=155)					
1. Behavioral intention	-	0.828	0.810	0.892	0.357
2. Automation trust		-	0.804	0.895	0.488
3. Competence			-	0.896	0.338
4. Autonomy				-	0.427
5. Relatedness					-

### 3.2. Descriptive statistics and analysis

**Figure 5:**
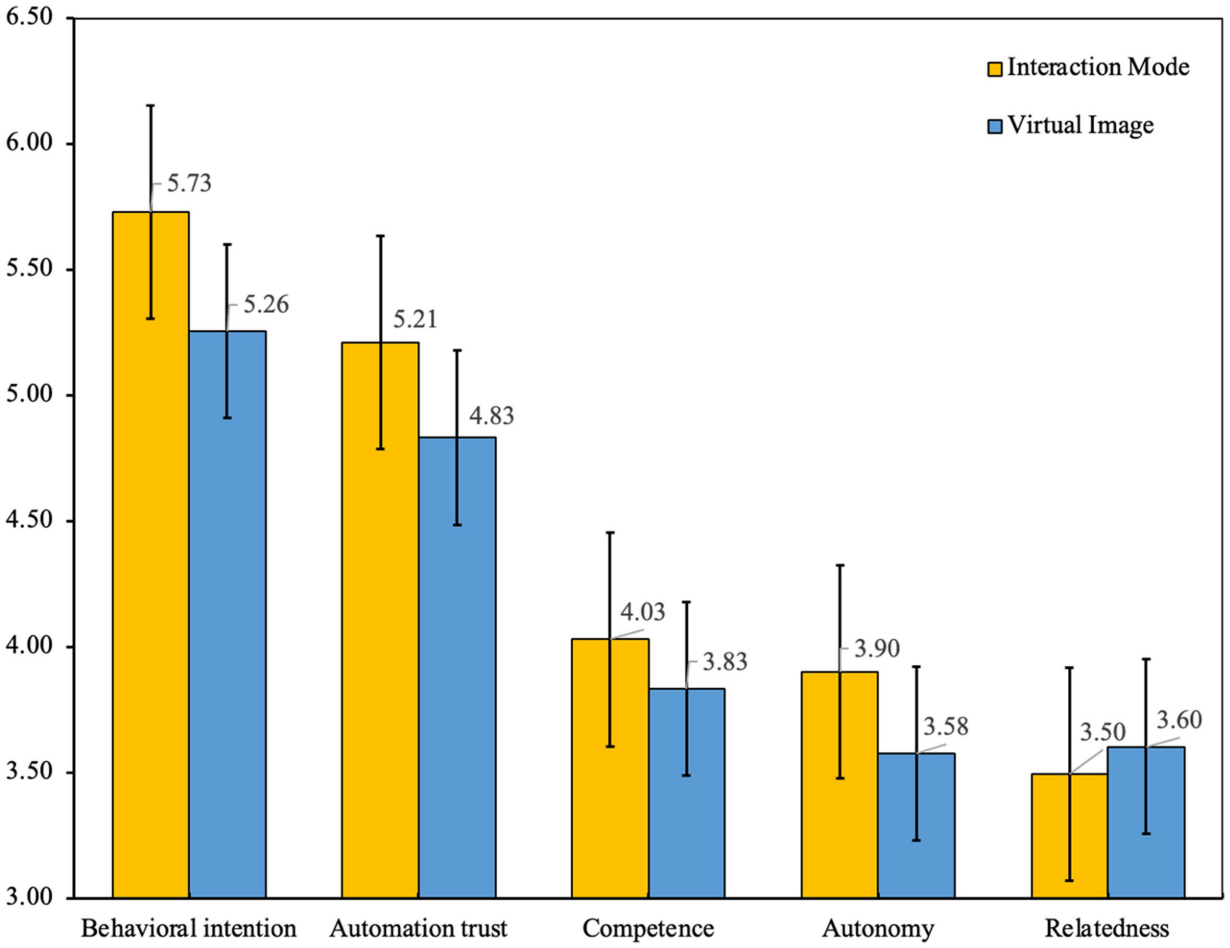
Mean score chart of the interaction mode and virtual image. Note: The scales for behavioral intention and automation trust were measured using a 7-point Likert scale, and the scales of competence, autonomy and relatedness were measured using a 5-point Likert scale.

Figure 5 shows the mean values of each measured variable for the interaction mode and virtual image. Regarding the overall consideration, the mean values of the scales indicate that the participants had a relatively high positive intention (*M* = 5.492, SD = 1.179) and trust (*M* = 5.021, SD = 1.045) in using the interaction mode and virtual image of AVs. Additionally, the participants had slightly positive attitudes toward the interaction mode and virtual image competence (*M* = 3.932, SD = 0.676), autonomy (*M* = 3.736, SD = 0.707), and relatedness (*M* = 3.550, SD = 0.858). Separately, an independent samples *t* test was used to test whether the behavioral intention and other test variables differed between the interaction technology (interaction mode and virtual image). Compared with the virtual image, the participants were significantly more willing to use an interaction mode [*t*(308) = −3.608, *p* = 0.000; M_IM_ = 5.729, SD_IM_ = 0.986; M_VI_ = 5.255, SD_VI_ = 1.306], perceived that the interaction mode should be more trusting [*t*(308) = −3.230, *p* = 0.001; M_IM_ = 5.210, SD_IM_ = 0.992; M_VI_ = 4.832, SD_VI_ = 1.066] and perceived that the interaction mode should be more focused on meeting the psychological needs of competence [*t*(308) = −2.577, *p* = 0.010*; M_IM_ = 4.030*, SD_IM_ = 0.601; M_VI_ = 3.834, SD_VI_ = 0.732] and autonomy [*t*(308) = −4.138, *p* = 0.000; M_IM_ = 3.898, SD_IM_ = 0.715; M_VI_ = 3.574, SD_VI_ = 0.662]. The perception of relatedness of the virtual image is higher than the interaction mode [*t*(308) = 1.126, *p* = 0.261; M_IM_ = 3.495, SD_IM_ = 0.862; M_VI_ = 3.604, SD_VI_ = 0.853, insignificant]. In terms of demographic measures, gender, driving duration and autonomous experience showed less of a relationship among the main measure variables. Therefore, we will not consider them in the next analysis.

### 3.3. Predicting users’ intention to use interaction technology

We used hierarchical multiple linear regression analyzes to assess, for each interaction technology, the contributions of the measured variables to the prediction of behavioral intention. Correlational analyzes indicated that gender, driving duration, and autonomous experience were not significant variables for behavioral intention, so they were not included as predictors. The results reflect the relationships among the three basic psychological needs, automation trust, and behavioral intention (obtained by regressing the predictive components of intention to use interaction technology) in Table 7.

**Table 7:** Hierarchical regression analyzes: predicting intention to use interaction technology.

Step and predictor	Interaction mode (*n* = 155)		Virtual image (*n* = 155)	
	Step 1 *β*	Step 2 *β*	Step 1 *β*	Step 2 *β*
Competence	0.243^**^	0.162^*^	0.224^**^	0.155
Autonomy	0.320^***^	0.239^***^	0.588^***^	0.388^***^
Relatedness	0.418^***^	0.350^***^	0.034	0.019
Automation trust		0.233^**^		0.335^***^
*R* ^2^	0.677	0.693	0.628	0.660
Δ*R*^2^	0.677	0.017	0.628	0.032
*F*_change_	105.29^***^	8.120^***^	84.85^***^	14.21^***^
Degree of change freedom	(3,151)	(1,150)	(3,151)	(1,150)

*p < 0.05, **p < 0.01, ***p < 0.001.

For each interaction technology, the critical predictors of participants’ intentions were identified by regressing the psychological needs of self-determination theory on behavioral intention and then adding automation trust to the regression model. This allowed us to control for the effects of other variables and assess the predictive role of each variable. Considering the interaction mode, in step 1, the three psychological needs (competence, autonomy, and relatedness) predictors were able to explain 67.7% of the behavioral intention [*F*(3,151) = 105.29, *p* = 0.000], and all three psychological needs had a significant effect on the willingness to use the interaction modality. In step 2, when automation trust was added to the regression analysis, it was able to lead to a significant increase in the degree of explanation to 69.3% [*F_change_* (1,150) = 8.120, *p* = 0.000]. All three psychological needs and automated trust became significant predictors (especially autonomy and relatedness), while competence decreased to a weakly significant level. Considering the virtual image, in step 1, the three psychological needs predictors were able to explain 62.8% of the behavioral intentions [*F*(3,151) = 84.85, *p* = 0.000], and the competence and autonomy of the three psychological needs had a significant effect on the intention to use the virtual image. The relatedness had no significant effect on the behavioral intention. Adding automation trust to the regression analysis led to a significant increase in the degree of explanation to 66.0% [*F_change_* (1,150) = 14.21, *p* = 0.000]. Autonomy and automation trust became highly significant predictors. The effect of competence diminished to the point of insignificance. Relatedness did not emerge as a significant predictor of behavioral intention. Therefore, we proved H1, H2, and H3.

A regression analysis was conducted to verify the role that automation trust plays in predicting behavioral intention for the three psychological needs and to test the corresponding hypotheses. For each of the two contexts, competence, autonomy, and relatedness were added to the regression variables, and the predictor variable was automation trust. This approach allowed us to the test associations among automation trust and the other variables studied by controlling for the effects of other variables. The results are summarized in Table 8. As shown in Table 8, for both interaction technologies, competence, autonomy, and relatedness all significantly affected trust. The adjusted R-squared values were 69.4% (interaction mode) and 71.3% (virtual image). The model also has a significant fitting effect [*F_IM_* (3,151) = 114.08, *p* = 0.000 and *F_VI_* (3,151) = 125.05, *p* = 0.000]. H4 has been proven. To confirm the mediating effect of automation trust, we performed bootstrap analyzes. For the interaction mode, the results of the bias-corrected percentile bootstrap method showed that the 95% BootCI in each group does not contain zero [95% BootCI_competence = (0.004, 0.175); 95% BootCI_autonomy = (0.006, 0.190); 95% BootCI_relatedness = (0.008, 0.194)]. Combining the results of the regression analysis, we concluded that automation trust partially mediated the prediction of the three basic psychological needs of SDT on behavioral intention. For the virtual image, the results of the bias-corrected percentile bootstrap method showed that the 95% BootCI in each group does not contain zero [95% BootCI_competence = (0.002, 0.162); 95% BootCI_autonomy = (0.080, 0.345); 95% BootCI_relatedness = (0.012, 0.114)]. Combining the regression analysis results, we concluded that automation trust has a partial mediating effect in the prediction of autonomy on behavioral intention and a full mediating effect in the prediction of relatedness and competence on behavioral intention. Then, H5, H6 and H7 were proven.

**Table 8:** Multiple linear regression: predicting the automation trust.

Predictor	Interaction mode	Virtual image
Competence	0.348^***^	0.207^***^
Autonomy	0.345^***^	0.599^***^
Relatedness	0.290^***^	0.158^***^
*R* ^2^	0.694	0.713
*F*	114.08^***^	125.05^***^
Degree of change freedom	(3,151)	(3,151)

*p < 0.05, **p < 0.01, ***p < 0.001.

Overall, for the interaction mode, autonomy, relatedness, and automation, trust was a strong determinant of behavioral intention, while competence was a vital subdeterminant. Autonomy and automation trust were the strongest predictors for virtual image, while competence and relatedness became the second most important predictors. Furthermore, automation trust mediates the predictive power of behavioral intention, and relatedness and competence indirectly influence behavioral intention by affecting automation trust.

## 4. Discussion

The goal of this research was to test the factors predicting users’ behavioral intentions to use interaction technology (interaction mode and virtual image) in AVs and to clarify the effect of self-determination theory and automation trust in the prediction model (especially for different interaction technologies). The data collected from the participants were utilized to test the proposed research model. The results showed that users’ behavioral intentions were affected by perceived autonomy, competence, relatedness, and automation trust. The proposed research model explained 69.3 and 66.0% of the total variance in users’ intentions in the interaction mode and virtual image, respectively. In addition, there were some differences in predicting users’ intentions due to the characteristics of the interaction technology.

From the perspective of psychological motivation theory, we examined the effects of perceived competence, autonomy, and relatedness on the interaction technology of AVs through the proposed research model. The findings suggest that perceived autonomy is considered a core predictor when predicting users’ intentions toward interaction technology, especially for the interaction mode. This finding is consistent with previous findings from Thongmak (2021), indicating that the predictive role of perceived autonomy on behavioral intention is straightforward. The hypothesis is confirmed. Perceived competence became a significant predictor of intention to use the interaction technology, showing a significant effect for different interaction technologies when making predictions about the intention to use. However, the direct predictive effect of perceived competence on behavioral intention was diminished after adding the automated trust variable, especially for virtual images. Perceived relatedness had a significant direct predictive effect on the intention to interact but not on the intention to use virtual images.

In terms of automation trust, the study findings show that automation trust is influenced by perceived competence, autonomy, and relatedness and directly affects behavioral intention, which is consistent with the hypothesis. Thus, the three psychological demands of SDT influence behavioral intention in two ways: directly and indirectly through automation trust. Furthermore, automation trust showed a significant positive effect in interaction mode and virtual image, which is an important predictor of intention to use. Verberne et al. (2015) also mentioned this conclusion. Furthermore, we demonstrate the mediating effect of automation trust. The mediating effect is not the same in the interaction mode and virtual image. This finding may be related to the characteristics of the interaction technology. The intention to use virtual images was directly influenced by automation trust and autonomy and indirectly influenced by relatedness and competence compared to the interaction mode. This indicates that people want virtual images to be well established and easier to use so they can trust them more to enhance their willingness to use them.

### 4.1. Theoretical implications

The promotion of new interaction media for AVs usually faces the problem of unclear users’ willingness and motivation to use them. In the context of the gradual commercialization of AVs and the rising usage of intelligent driving interaction technologies, it is of great practical significance to understand users’ motivations to use new interaction technologies for AVs, improve their design, and increase user satisfaction and acceptance. Many scholars have meaningfully explored this proposition, but the influence mechanisms explaining users’ behavioral intentions from psychological motivation and cognitive perspectives have not been fully developed. Our research provided more meaningful results in this area.

First, this study explored the suitable measurement dimensions of psychological motivation for observing new interaction technologies and discussed the mechanisms of psychological motivation influencing behavioral intention for different interaction technologies. Our results are consistent with previous findings on the impact of technological trust and self-determined motivation on intentions to use wearable fitness technology (Rupp et al., 2016), indicating that perceived autonomy is a strong predictor of acceptance intention in SDT. Perceived competence and relatedness reflect users’ feelings before and after use. Although the predictive power of these three factors varies across contexts, the association between them and the intention to use AV interaction technology is strong. These findings may help advance the literature on the application of SDT in autonomous vehicles and provide new foundations and research directions for scholars and researchers to further expand the existing body of knowledge.

Second, among the seriousness related to behavioral intention regarding AVs, the level of automation trust was considered a key factor in deciding whether to use AVs. Based on this consideration, this study further explored the predictive role of automation trust on behavioral intention and the mediating role it plays in the relationship between psychological motivation and behavioral intention. This approach includes driving safety and positive experience in the same evaluation system, which allows researchers to gain a comprehensive understanding of users’ behavioral intentions regarding AV interaction technology.

Third, our results show that context-specific differences influence user acceptance of the interaction medium of AVs. For example, perceived autonomy, perceived competence, and automation trust jointly determine the behavioral intention to interact with virtual images. However, in the interaction mode, perceived autonomy plays a more important role than automation trust in explaining the user’s behavioral intentions. These findings help researchers understand the relationships among users’ psychological motivations and the characteristics of interaction techniques.

### 4.2. Practical implications

In addition to theoretical implications, this study provides valuable guidance for the design and dissemination of intelligent interaction technologies for AVs.

First, we found a significant role for autonomy in the prediction of users’ behavioral intentions for both interaction technologies. This finding suggests that users are motivated to use the interaction technology for the independence provided by using that service for a given interaction. This result may not be consistent with the philosophy of most AV designers and manufacturers. AV designers generally believe that they should improve the functionality and smartness of their products to allow the systems to satisfy humans’ needs automatically. They often design automated systems such as automatically starting and stopping the air conditioner, opening the sunroof, recommending music, etc. However, the research results show that users do not seem to accept this automation at this stage. We need to give users some autonomy and decision opportunity.

Second, we cannot ignore the important role of automation trust. At this stage, there are still discussions about safety and competence in users’ perceptions of AVs, and users still have doubts about their capabilities. This required that we should pay attention to avoid errors and failures in users’ use. Designers need to improve the stability and reliability of the technology to build user trust in the technology. In addition, we should improve public perceptions of the technology and encourage more users to trust AVs. This measure may result in consumers being more willing to use the technology and having more psychological needs met while using it.

Third, we cannot ignore the model differences in the impact of relatedness and competence on behavioral intentions for virtual images and interaction mode. This suggests the need for separate discussion and design of modules and functions directly related to user use when predicting user acceptance of AVs. Users’ attitudes differ at the functional level.

### 4.3. Limitations and further research directions

The current study has several limitations that warrant further consideration. First, the study investigated drivers’ behavioral intentions to adopt the new interaction technology brought by autonomous vehicles but not drivers’ intentions to use autonomous vehicles. The positive attitude toward the new interaction technology might be shifted to autonomous vehicles. Second, the process of influence on behavioral intentions is extremely complex. We focused only on behavioral intentions at first contact and understanding. In the future, we should pay attention to the dynamic adjustment process of behavioral intentions. Finally, our selected sample focused on middle-aged and young adults aged 20–40 years. In future research, we should pay attention to whether older people accept autonomous vehicles and the new interaction technology differently than younger people.

## 5. Conclusion

This study investigated drivers’ behavioral intentions to adopt the new interaction technology brought by autonomous vehicles: interaction mode and virtual image. The following conclusions were reached. First, as the results of this study show, perceived autonomy and automation trust are important factors that directly influence consumers’ behavioral intentions to adopt AV interaction mode and virtual image. Automation trust for the interaction mode and virtual image was significantly influenced by perceived autonomy, relatedness, and competence. Second, in the interaction mode, perceived relatedness and perceived competence directly and significantly affected behavioral intention. However, in virtual images, this direct effect is much weaker. Third, in addition to the direct effects of the measured variables, the three psychological demands also affect behavioral intention by influencing automation trust for interaction mode and virtual image. This effect is strongest for the virtual image. The mediating role played by automation trust in the model can be clarified. This information will help AV design, interaction mode settings, and drivers’ personalized designs. This can guide the use of suitable interaction technology for improving customers’ use intentions.

## Data availability statement

The raw data supporting the conclusions of this article will be made available by the authors, without undue reservation.

## Ethics statement

The studies involving human participants were reviewed and approved by the Human Research Ethics Committee of the School of Economics and Management, Beihang University. The patients/participants provided their written informed consent to participate in this study.

## Author contributions

RZ conceived the study. YX, MQ, and MJ contributed to the experimental execution and data collection. RZ and YX contributed to data curation, formal analysis, and validation. YX and AC contributed to writing paper drafts. RZ and AC contributed to supervision. All authors contributed to the article and approved the submitted version.

## Funding

This study was supported by the National Natural Science Foundation of China (NSFC, 72171015 and 72021001).

## Conflict of interest

The authors declare that the research was conducted in the absence of any commercial or financial relationships that could be construed as a potential conflict of interest.

## Publisher’s note

All claims expressed in this article are solely those of the authors and do not necessarily represent those of their affiliated organizations, or those of the publisher, the editors and the reviewers. Any product that may be evaluated in this article, or claim that may be made by its manufacturer, is not guaranteed or endorsed by the publisher.

## References

[ref1] Acheampong R. A. Cugurullo F. (2019). Capturing the determinants behind the adoption of autonomous vehicles: conceptual frameworks and measurement models to predict public transport, sharing and ownership trends of self-driving cars. Transport. Res. F: Traffic Psychol. Behav. 62, 349–375. doi: 10.1016/j.trf.2019.01.009

[ref2] Adnan N. Nordin S. M. Bin Bahruddin M. A. Ali M. (2018). How trust can drive forward the user acceptance to the technology? In-vehicle technology for autonomous vehicle. Transp. Res. A Policy Pract. 118, 819–836. doi: 10.1016/j.tra.2018.10.019

[ref4] Bansal P. Kockelman K. M. (2017). Forecasting Americans’ long-term adoption of connected and autonomous vehicle technologies. Transp. Res. A Policy Pract. 95, 49–63. doi: 10.1016/j.tra.2016.10.013

[ref95] Barber B. (1983). The logic and limits of trust.

[ref6] Bazilinskyy P. Kyriakidis M. de Winter J. (2015). An international crowdsourcing study into people's statements on fully automated driving. Proc. Manuf. 3, 2534–2542. doi: 10.1016/j.promfg.2015.07.540

[ref7] Bimbraw K. (2015). Autonomous Cars: Past, Present and Future a Review of the Developments in the Last Century, The Present Scenario and the Expected Future of Autonomous Vehicle Technology. In *2015 12th International Conference on Informatics in Control, Automation and Robotics (ICINCO)*. Piscataway: IEEE, pp. 191–198.

[ref8] Buckley L. Kaye S. A. Pradhan A. K. (2018). Psychosocial factors associated with intended use of automated vehicles: a simulated driving study. Accid. Anal. Prev. 115, 202–208. doi: 10.1016/j.aap.2018.03.021, PMID: 29631216

[ref9] Chen S. C. Chen H. H. Chen M. F. (2009). Determinants of satisfaction and continuance intention towards self-service technologies. Ind. Manag. Data Syst. 109, 1248–1263. doi: 10.1108/02635570911002306

[ref10] Chiou E. K. Lee J. D. (2021). Trusting automation: designing for responsivity and resilience. Hum. Factors 65, 137–165. doi: 10.1177/00187208211009995, PMID: 33906505

[ref12] Choi J. K. Ji Y. G. (2015). Investigating the importance of trust on adopting an autonomous vehicle. Int. J. Hum. –Comput. Interact. 31, 692–702. doi: 10.1080/10447318.2015.1070549

[ref14] Comrey A. L. Lee H. B. (2013). A First Course in Factor Analysis. London: Psychology Press.

[ref15] Davis F. D. (1989). Perceived usefulness, perceived ease of use, and user acceptance of information technology. MIS Q. 13, 319–340. doi: 10.2307/249008

[ref90] Deci E. L. Connell J. P. Ryan R. M. (1989). Self-determination in a work organization. Journal of applied psychology, 74, 580. doi: 10.1037/0021-9010.74.4.580

[ref16] Deci E. L. Ryan R. M. (1991). A motivational approach to self: integration in personality. Nebr. Symp. Motiv. 38, 237–288. 2130258

[ref18] Deci E. L. Ryan R. M. (2012). The Oxford Handbook of Human Motivation. Oxford: Oxford University Press.

[ref20] Fornell C. Larcker D. (1981). Evaluating structural equation models with unobservable variables and measurement error: algebra and statistics. J. Mark. Res. 18, 382–388. doi: 10.1177/002224378101800313

[ref21] Fuyun L. Jianping F. Mousong F. (2019). A Natural Human-Computer Interaction Method In *Virtual Roaming. In 2019 15th International Conference on Computational Intelligence and Security (CIS)*. Piscataway: IEEE, pp. 411–414.

[ref22] Grouzet F. M. Kasser T. Ahuvia A. Dols J. M. F. Kim Y. Lau S.. (2005). The structure of goal contents across 15 cultures. J. Pers. Soc. Psychol. 89, 800–816. doi: 10.1037/0022-3514.89.5.800, PMID: 16351369

[ref23] Hair J. F. Jr. Hult G. T. M. Ringle C. M. Sarstedt M. Danks N. P. Ray S. (2021). Partial Least Squares Structural Equation Modeling (PLS-SEM) Using R: A Workbook, Berlin: Springer.

[ref24] Hensher D. A. (2018). Tackling road congestion–what might it look like in the future under a collaborative and connected mobility model? Transp. Policy 66, A1–A8. doi: 10.1016/j.tranpol.2018.02.007

[ref26] Hergenhahn B. R. Henley T. (2013). An Introduction to the History of Psychology. Boston: Cengage Learning.

[ref85] Hew J. J. Badaruddin M. N. B. A. Moorthy M. K. (2017). Crafting a smartphone repurchase decision making process: Do brand attachment and gender matter?. Telematics and Informatics, 34, 34–56. doi: 10.1016/j.tele.2016.12.009

[ref27] Hoffman D. L. Novak T. (2012). Why do people use social media? Empirical findings and a new theoretical framework for social media goal pursuit. SSRN Electron. J. doi: 10.2139/ssrn.1989586

[ref28] Huang Y. C. Backman S. J. Backman K. F. McGuire F. A. Moore D. (2019). An investigation of motivation and experience in virtual learning environments: a self-determination theory. Educ. Inf. Technol. 24, 591–611. doi: 10.1007/s10639-018-9784-5

[ref29] Jian J. Y. Bisantz A. M. Drury C. G. (2000). Foundations for an empirically determined scale of trust in automated systems. Int. J. Cogn. Ergon. 4, 53–71. doi: 10.1207/S15327566IJCE0401_04

[ref30] Jung Y. (2011). Understanding the role of sense of presence and perceived autonomy in users' continued use of social virtual worlds. J. Comput.-Mediat. Commun. 16, 492–510. doi: 10.1111/j.1083-6101.2011.01540.x

[ref31] Jung Y. Perez-Mira B. Wiley-Patton S. (2009). Consumer adoption of mobile TV: examining psychological flow and media content. Comput. Hum. Behav. 25, 123–129. doi: 10.1016/j.chb.2008.07.011

[ref32] Kaur K. Rampersad G. (2018). Trust in driverless cars: investigating key factors influencing the adoption of driverless cars. J. Eng. Technol. Manag. 48, 87–96. doi: 10.1016/j.jengtecman.2018.04.006

[ref33] Kim Y. H. Kim D. J. (2005). A Study of Online Transaction Self-Efficacy, Consumer Trust, and Uncertainty Reduction in Electronic Commerce Transaction. In *Proceedings of the 38th Annual Hawaii International Conference on System Sciences*. Piscataway: IEEE, pp. 170c.

[ref34] Koole S. L. Schlinkert C. Maldei T. Baumann N. (2019). Becoming who you are: an integrative review of self-determination theory and personality systems interactions theory. J. Pers. 87, 15–36. doi: 10.1111/jopy.12380, PMID: 29524339 PMC6378399

[ref105] Kofi Frimpong A. N. Li, P. Nyame G. Hossin M. A. (2020). The Impact of Social Media Political Activists on Voting Patterns. Political Behavior, 44, 599–652. doi: 10.1007/s11109-020-09632-3

[ref35] Lee J. Lee D. Park Y. Lee S. Ha T. (2019). Autonomous vehicles can be shared, but a feeling of ownership is important: examination of the influential factors for intention to use autonomous vehicles. Transp. Res. C Emerg. Technol. 107, 411–422. doi: 10.1016/j.trc.2019.08.020

[ref36] Lee J. D. See K. A. (2004). Trust in automation: designing for appropriate reliance. Hum. Factors 46, 50–80. doi: 10.1518/hfes.46.1.50.30392, PMID: 15151155

[ref37] Li T. Gupta S. Zhou H. (2021). An empirical study on drivers’ willingness to use automatic features of intelligent vehicles: a psychological empowerment perspective. Front. Psychol. 12:794845. doi: 10.3389/fpsyg.2021.794845, PMID: 34975696 PMC8716813

[ref38] Liu L. Liu J. (2021). Research on Image Design of Chinese Characters in Virtual Reality. In *International Conference on Artificial Intelligence, Virtual Reality, and Visualization (AIVRV 2021), Volume 12153*. Bellingham: SPIE, pp. 181–185.

[ref40] Long S. K. Sato T. Millner N. Loranger R. Mirabelli J. Xu V.. (2020). Empirically and Theoretically Driven Scales on Automation Trust: A Multi-Level Confirmatory Factor Analysis. In *Proceedings of the Human Factors and Ergonomics Society Annual Meeting, Volume 64*. Los Angeles, CA: SAGE Publications, pp. 1829–1832.

[ref41] Lu N. Cheng N. Zhang N. Shen X. Mark J. W. (2014). Connected vehicles: solutions and challenges. IEEE Internet Things J. 1, 289–299. doi: 10.1109/JIOT.2014.2327587

[ref42] Lutin J. M. Kornhauser A. L. Masce E. L. L. (2013). The revolutionary development of self-driving vehicles and implications for the transportation engineering profession. ITE J. 83:28.

[ref43] Lyons J. B. Wynne K. T. Mahoney S. Roebke M. A. (2019). “Trust and human-machine teaming: a qualitative study” in Artificial Intelligence for the Internet of Everything (Amsterdam, Netherland: Elsevier), 101–116.

[ref44] Müller J. M. (2019). Comparing technology acceptance for autonomous vehicles, battery electric vehicles, and car sharing—a study across Europe, China, and North America. Sustainability 11:4333. doi: 10.3390/su11164333

[ref45] Murali P. K. Kaboli M. Dahiya R. (2022). Intelligent in-vehicle interaction technologies. Adv. Intell. Syst. 4:2100122. doi: 10.1002/aisy.202100122

[ref46] Natarajan M. Gombolay M. (2020). Effects of Anthropomorphism and Accountability on Trust in Human Robot Interaction. In *Proceedings of the 2020 ACM/IEEE International Conference on Human-Robot Interaction*, pp. 33–42.

[ref47] Niu D. Terken J. Eggen B. (2018). “Anthropomorphizing information to enhance trust in autonomous vehicles” in Human Factors and Ergonomics in Manufacturing and Service Industries. ed. Salmon P., vol. 28, 352–359.

[ref48] Parasuraman R. Riley V. (1997). Humans and automation: Use, misuse, disuse, abuse. Hum. Factors 39, 230–253. doi: 10.1518/001872097778543886

[ref49] Peters D. Calvo R. A. Ryan R. M. (2018). Designing for motivation, engagement and wellbeing in digital experience. Front. Psychol. 9:797. doi: 10.3389/fpsyg.2018.00797, PMID: 29892246 PMC5985470

[ref51] Racero F. J. Bueno S. Gallego M. D. (2020). Predicting students’ behavioral intention to use open source software: a combined view of the technology acceptance model and self-determination theory. Appl. Sci. 10:2711. doi: 10.3390/app10082711

[ref52] Rödel C. Stadler S. Meschtscherjakov A. Tscheligi M. (2014). Towards Autonomous Cars: The Effect of Autonomy Levels on Acceptance and User Experience. In *Proceedings of the 6th International Conference on Automotive user Interfaces and Interactive Vehicular Applications*, pp. 1–8.

[ref54] Rupp M. A. Michaelis J. R. McConnell D. S. Smither J. A. (2016). The impact of technological trust and self-determined motivation on intentions to use wearable fitness technology. In *Proceedings of the Human Factors and Ergonomics Society Annual Meeting, Volume 60*. Los Angeles, CA: Sage Publications, pp. 1434–1438.

[ref55] Ryan M. (2020). The future of transportation: ethical, legal, social and economic impacts of self-driving vehicles in the year 2025. Sci. Eng. Ethics 26, 1185–1208. doi: 10.1007/s11948-019-00130-2, PMID: 31482471 PMC7286843

[ref56] Ryan R. M. Deci E. L. (2000a). Self-determination theory and the facilitation of intrinsic motivation, social development, and well-being. Am. Psychol. 55, 68–78. doi: 10.1037/0003-066X.55.1.68, PMID: 11392867

[ref57] Ryan R. M. Deci E. L. (2000b). Intrinsic and extrinsic motivations: classic definitions and new directions. Contemp. Educ. Psychol. 25, 54–67. doi: 10.1006/ceps.1999.1020, PMID: 10620381

[ref58] Ryan R. M. Deci E. L. (2017). Self-Determination Theory: Basic Psychological Needs in Motivation, Development, and Wellness. New York: Guilford Publications.

[ref100] Sadi M. A. Al-Khalifah A. M.. (2012). Factors influencing trust in on-line shopping: a case of Saudi Arabian consumer behavior. Journal of Emerging Trends in Economics and Management Sciences, 3, 517–522. https://hdl.handle.net/10520/EJC127666

[ref60] Schaefer K. E. Billings D. R. Szalma J. L. Adams J. K. Sanders T. L. Chen J. Y.. (2014). *A Meta-analysis of Factors Influencing the Development of Trust in Automation: Implications for Human-robot Interaction*. Army Research Lab Aberdeen Proving Ground Md Human Research and Engineering Directorate.

[ref61] Seuwou P. Chrysoulas C. Banissi E. Ubakanma G. (2020). Measuring Consumer Behavioural Intention to Accept Technology: Towards Autonomous Vehicles technology acceptance model (AVTAM). In *World Conference on Information Systems and Technologies*. Springer: Cham, 507–516.

[ref63] Sotolongo N. Copulsky J. (2018). Conversational marketing: creating compelling customer connections. Appl. Mark. Anal. 4, 6–21.

[ref64] Tabachnick B. G. Fidell L. S. Ullman J. B. (2007). Using Multivariate Statistics. 5. Boston, MA: Pearson, 481–498.

[ref65] Tan Z. Dai N. Su Y. Zhang R. Li Y. Wu D.. (2021). Human-machine interaction in intelligent and connected vehicles: a review of status quo, issues and opportunities. IEEE Trans. Intell. Transp. Syst. 23, 13954–13975. doi: 10.1109/TITS.2021.3127217

[ref66] Thongmak M. (2021). A model for enhancing employees’ lifelong learning intention online. Learn. Motiv. 75:101733. doi: 10.1016/j.lmot.2021.101733

[ref67] Tyack A. Mekler E. D. (2020). Self-Determination Theory in HCI Games Research: Current Uses and Open Questions. In *Proceedings of the 2020 CHI Conference on Human Factors in Computing Systems*, pp. 1–22.

[ref68] Velasco J. P. N. Farah H. van Arem B. Hagenzieker M. P. (2019). Studying pedestrians’ crossing behavior when interacting with automated vehicles using virtual reality. Transport. Res. F: Traffic Psychol. Behav. 66, 1–14. doi: 10.1016/j.trf.2019.08.015

[ref69] Venkatesh V. Davis F. D. (2000). A theoretical extension of the technology acceptance model: four longitudinal field studies. Manag. Sci. 46, 186–204. doi: 10.1287/mnsc.46.2.186.11926

[ref70] Venkatesh V. Thong J. Y. Xu X. (2012). Consumer acceptance and use of information technology: extending the unified theory of acceptance and use of technology. MIS Q. 36:157. doi: 10.2307/41410412

[ref71] Verberne F. M. Ham J. Midden C. J. (2015). Trusting a virtual driver that looks, acts, and thinks like you. Hum. Factors 57, 895–909. doi: 10.1177/0018720815580749, PMID: 25921302

[ref72] Villalobos-Zúñiga G. Cherubini M. (2020). Apps that motivate: a taxonomy of app features based on self-determination theory. Int. J. Hum. Comput. Stud. 140:102449. doi: 10.1016/j.ijhcs.2020.102449

[ref73] Wang K. (2021). Human-computer interaction design of intelligent vehicle-mounted products based on the internet of things. Mob. Inf. Syst. 2021, 1–12. doi: 10.1155/2021/6795440

[ref74] Wang X. Li Y. (2014). Trust, psychological need, and motivation to produce user-generated content: a self-determination perspective. J. Electron. Commer. Res. 15, 241–253.

[ref75] Yang X. Aurisicchio M. (2021). Designing Conversational Agents: A Self-determination Theory Approach. In *Proceedings of the 2021 CHI Conference on Human Factors in Computing Systems*, pp. 1–16.

[ref76] Yuen K. F. Cai L. Qi G. Wang X. (2021). Factors influencing autonomous vehicle adoption: an application of the technology acceptance model and innovation diffusion theory. Tech. Anal. Strat. Manag. 33, 505–519. doi: 10.1080/09537325.2020.1826423

[ref77] Zhang T. Tao D. Qu X. Zhang X. Lin R. Zhang W. (2019). The roles of initial trust and perceived risk in public’s acceptance of automated vehicles. Transp. Res. C Emerg. Technol. 98, 207–220. doi: 10.1016/j.trc.2018.11.018

[ref78] Zheng P. Wang Z. Chen C. H. Khoo L. P. (2019). A survey of smart product-service systems: key aspects, challenges and future perspectives. Adv. Eng. Inform. 42:100973. doi: 10.1016/j.aei.2019.100973

[ref79] Zhou J. Luo S. Chen F. (2020). Effects of personality traits on user trust in human–machine collaborations. J. Multimodal User Interfaces 14, 387–400. doi: 10.1007/s12193-020-00329-9

[ref80] Ziyan C. Shiguo L. (2021). China's self-driving car legislation study. Comput. Law Secur. Rev. 41:105555. doi: 10.1016/j.clsr.2021.105555

